# Characterization of Hydrolytic and Thermomechanical Stability of 3D-Printed PLDLA-TMC 60/40 Scaffolds for Cartilage Tissue Applications

**DOI:** 10.1021/acsomega.5c06554

**Published:** 2026-02-13

**Authors:** Flavia Pedrini, Ariana S. Moraes, Bruna V. Quevedo, Moema A. Hausen, Rodrigo C. Gomes, Daniel Komatsu, Eliana A. R. Duek

**Affiliations:** † 600424 Federal University of São Carlos, Center of Sciences and Technologies for Sustainability, Postgraduate Program in Biotechnology and Environmental Monitoring (PPGBMA), Sorocaba 18.052-780, Brazil; ‡ 67780 Pontifical Catholic University of São Paulo, Faculty of Medical Sciences and Health, Surgery Department, Postgraduate Program in Biomaterials and Regenerative Medicine (PPGBMR), Sorocaba 18.030-070, Brazil; § 600424 Federal University of São Carlos, Center of Sciences and Technologies for Sustainability, Postgraduate Program in Materials Sciences (PPGCM), Sorocaba 18.052-780, Brazil; ∥ Pontifical Catholic University of São Paulo (PUC-SP), Faculty of Medical Sciences and Health (FCMS), Laboratory of Biomaterials, Sorocaba 18.030-070, Brazil

## Abstract

Biodegradable thermoplastic polymers are increasingly explored in regenerative medicine due to their potential to mimic native tissue environments. Among them, poly­(L-*co*-D,L-lactic acid-*co*-trimethylene carbonate) (PLDLA-TMC) offers tunable degradation and biocompatibility features. However, extrusion-based 3D printing may induce polymer chain scission, compromising scaffold integrity under physiological conditions. Although PLDLA-TMC has been explored in various biomedical applications, there is a lack of studies assessing its performance under mechanically stimulated environments that emulate *in vivo* conditions, which limits its translation to load-bearing tissues such as cartilage. To address this gap, this study investigates the hydrolytic and thermomechanical degradation of 3D-printed PLDLA-TMC 60/40 scaffolds and their biological behavior under dynamic perfusion culture. Temperature-dependent degradation was confirmed, as printing at 120 °C preserved polymer integrity more efficiently, while both *M*_n_ and *M*_w_ decreased by approximately 50% after 2 weeks of hydrolytic degradation and by 80% after 4 weeks (*p* < 0.01). Dynamic culture assays demonstrated enhanced chondrogenesis, with type II collagen and SOX9 showing approximately 2-fold higher fluorescence intensities. Although aggrecan displayed slightly higher total labeling under static culture, dynamic perfusion reduced the fluorescence peak width by 40%, supporting a more stratified hyaline-like matrix. Perfusion also promoted significantly greater cell infiltration (*p* < 0.05). Altogether, these results highlight the suitability of PLDLA-TMC scaffolds for mechanically stimulated tissue-engineering applications, particularly cartilage regeneration.

## Graphical abstract

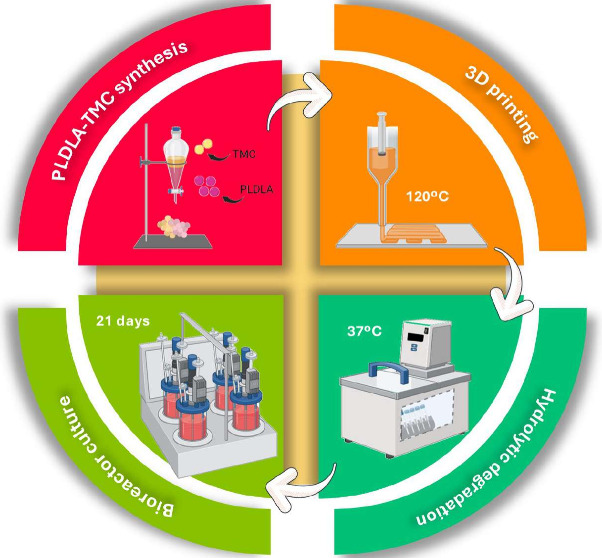

## 1 Introduction

Over the past decades, the pursuit of advanced materials for tissue engineering has driven growing interest in biodegradable thermoplastic polymers, particularly due to their favorable interactions with biological systems and tunable degradation profiles.^1^ Among these, aliphatic polyesters derived from lactic acid stand out for their biocompatibility, controlled resorption, and processing versatility. Poly­(L-*co*-D,L-lactic acid) (PLDLA), a copolymer of l-lactide and D,l-lactide, exhibits intermediate crystallinity and adjustable degradation kinetics, making it a suitable candidate for diverse regenerative applications.^2–5^ However, despite these advantages, PLDLA remains mechanically fragile and lacks the elasticity required for use in soft tissues or dynamically loaded environments.^6^

To address these limitations, the incorporation of flexible monomer units such as trimethylene carbonate (TMC) has been explored.^7,8^ By adjusting the ratio between PLDLA and TMC, it is possible to modulate the physicochemical, thermal, rheological, and mechanical properties of the resulting terpolymer, allowing its performance to be tailored for specific applications. In fact, a previous study demonstrated that varying the PLDLA-to-TMC ratio significantly affects the mechanical behavior of the material. Specifically, when Young’s modulus was evaluated, scaffolds composed of PLDLA-TMC at a 60/40 ratio exhibited a marked increase in flexibility compared to other formulations.^9^ Beyond improved flexibility, the PLDLA-TMC terpolymer exhibits enhanced ductility and a more uniform degradation profile, making it particularly promising for applications where mechanical compliance and controlled bioabsorption are essential. Its hydrolytic degradation, driven by the cleavage of ester bonds in aqueous environments, is influenced by factors including composition, crystallinity, and molecular weight.^10^ The inclusion of TMC increases the hydrophilicity and amorphous nature of the polymer, facilitating water penetration and accelerating degradation, an important attribute when tailoring scaffold resorption to match the pace of tissue development.^11,12^

Degradation behavior plays a central role in scaffold design, influencing both the mechanical stability of the construct and its biological integration.^13,14^ During extrusion-based 3D printing, the biomaterial is subjected to degradation due to mechanical and thermal stresses, which induce polymer chain scission.^15–17^ This process can lead to a reduction in molecular weight and alteration of material properties postprinting, underscoring the importance of understanding how manufacturing parameters affect scaffold performance.^18^ Despite the challenges introduced during fabrication, the printed scaffolds must still maintain their structural integrity and functional properties under physiological conditions.

Although PLDLA-TMC copolymers have been extensively studied for their tunable mechanical and degradation properties, there is still a limited understanding of how these materials behave after 3D printing and under dynamic culture conditions that better mimic physiological environments. Together, extrusion-induced degradation and perfusion-driven dynamic stimuli are expected to modulate the molecular and degradative evolution of PLDLA-TMC scaffolds, ultimately impacting their suitability for cartilage tissue engineering.

Accordingly, this study aims to investigate how extrusion-based processing influences the molecular and thermomechanical properties of PLDLA-TMC scaffolds and how these properties evolve during hydrolytic degradation. In parallel, it evaluates scaffold behavior under static and dynamic culture conditions to determine how perfusion-derived mechanical and mass-transport stimuli affect their structural and functional stability. Unlike conventional degradation studies, this work combines extrusion-based 3D printing with perfusion bioreactor culture to evaluate scaffold performance under physiologically relevant conditions. This strategy provides deeper insight into how fabrication-induced degradation and dynamic mechanical stimuli jointly influence scaffold integrity and functionality. Ultimately, this integrative approach contributes to bridging the gap between advanced polymer development, additive manufacturing, and dynamic *in vitro* modeling, providing a more predictive model of scaffold behavior *in vivo* and supporting the creation of next-generation scaffolds for cartilage tissue engineering.

## 2 Materials and Methods

The poly­(L-*co*-D,L-lactic acid-*co*-trimethylene carbonate) (PLDLA-TMC) pellets used for 3D printing were previously synthesized at a 60/40 ratio, and the monomers L-lactate (CAS number 4511–42–6), D,L-lactate (CAS number 95–96–5) was purchased from Purasorb Purac Biochem (Gorinchem, Netherlands). Trimethylene carbonate (TMC) (CAS 2453–03–4) was purchased from Boehringer Ingelheim (Ingelheim am Rhein, Germany).

### 2.1 3D Printing

Scaffolds were fabricated using a piston-driven extrusion bioprinter (Octopus, 3D Biotechnology Solutions, São Paulo, Brazil), without platform heating. Scaffold design (10 mm diameter × 2.4 mm thickness) and slicing parameters were defined in *Slic3r* software. Printing was conducted at temperatures ranging from 120 to 190 °C, in 10 °C intervals. A layer height of 0.4 mm and a printing speed of 4 mm/s were used, with a rectilinear infill pattern at 90% density. Printing was performed in open air at 25 °C.

### 2.2 Gel Permeation Chromatography (GPC)

Number-average molecular weight (*M*_n_), weight-average molecular weight (*M*_w_), and polydispersity index (PDI) were determined using a Waters GPC system (Waters Corporation, Milford, MA, USA) equipped with a 2414 refractive index detector. Analyses were carried out with tetrahydrofuran (THF) (HPLC grade, ≥ 99.9%, Sigma-Aldrich, #401757) as the mobile phase, at 35 °C, using two Styragel HR 5 μm columns (7.8 × 300 mm). Sample solutions (3.00 mg mL^–1^) were injected manually at a 1 mL min^–1^ flow rate. Calibration was performed using monodisperse polystyrene standards (Sigma-Aldrich).

### 2.3 Carbonyl Index

The carbonyl index was calculated based on the ratio between the intensity at the maximum point of the carbonyl band (C=O) at 1750 cm^–1^ and the intensity at the maximum point of the C–H band at 1453 cm-1,^19^ according to eq 1.

1 carbonyl
index=intensity of the
C=O band/intensity of the
C−H band

FT-IR spectra were obtained using a Spectrum 65 spectrometer (PerkinElmer, Waltham, MA, USA) equipped with an attenuated total reflection (ATR) accessory. Measurements were carried out over the spectral range of 4000 to 600 cm^–1^, with a resolution of 4 cm^–1^, averaging 32 scans per sample.

### 2.4 Thermal Analysis

#### 2.4.1 Differential Scanning Calorimetry (DSC)

DSC measurements were performed using a DSC 25 instrument (TA Instruments, New Castle, DE, USA). For the first heating cycle, 8 mg of the samples were analyzed in a temperature range from −50 to 200 °C, with a heating rate of 10 °C min^–1^ and a nitrogen flow of 20 mL min^–1^. Subsequently, the samples were cooled to −50 °C at a 10 °C min^–1^ rate and held at this temperature for 1 min. The samples were heated to 200 °C for the second heating cycle, using the same heating rate and nitrogen flow.

#### 2.4.2 Thermogravimetric Analysis (TGA)

The thermogravimetric analyses were performed on a TGA 55 equipment (TA Instruments, New Castle, DE, USA). The samples (8 mg) were analyzed in a temperature range from 25 to 400 °C, with a heating rate of 10 °C min^–1^ and a nitrogen flow of 100 mL min^–1^.

### 2.5 Hydrolytic Degradation

The *in vitro* degradation of the PLDLA-TMC 60/40 scaffolds was performed in phosphate-buffered saline (PBS) (Sigma-Aldrich, #P3813) at pH 7.4. Each scaffold was immersed in 10 mL of PBS in individual 15 mL polypropylene tubes and incubated in a thermostatic bath at 37 °C under static conditions for 2 and 4 weeks. The PBS was replaced every 3 days to maintain physiological ionic strength. Scaffolds subjected to hydrolytic degradation were analyzed in triplicate (n = 3). After the incubation periods, the samples were rinsed with deionized water, dried under vacuum at 25 °C for 48 h, and subsequently analyzed by GPC, DSC, TGA and scanning electron microscopy (SEM).

### 2.6 Scanning Electron Microscopy (SEM)

The scaffolds’ morphology was examined using a Quanta 250 SEM (FEI Company, Hillsboro, OR, USA). Samples were sputter-coated with a 30 nm gold layer using a Leica EM ACE200 sputter coater. Imaging was performed at 10 kV accelerating voltage.

### 2.7 Bioreactor Dynamic Culture

Mesenchymal stem cells (MSCs) were used for cell differentiation, acquired from Thermo Fisher Scientific (#R7788110). The cells, frozen in liquid nitrogen (−196 °C), were thawed in DMEM (Dulbecco’s Modified Eagle’s Medium, Sigma-Aldrich, #D6046), supplemented with 10% fetal bovine serum (FBS) (Sigma-Aldrich, #F1051) and antibiotics. The cells were expanded in 75 cm^2^ cell culture flasks, and after reaching approximately 90% confluence, they were trypsinized with a 0.2% trypsin-EDTA solution. Cells between the third and fifth passages were used in the experiments.

For MSC differentiation, the StemPro Kit (Gibco, #A1007101), supplemented with the chondrogenic inducers BMPs and TGF-β1, was used. The MSCs were seeded onto the PLDLA-TMC 60/40 scaffolds (10 mm in diameter by 2.4 mm in thickness) in a DMEM medium, supplemented with 10% FBS and antibiotics. Once the culture was established (4 days), the standard medium was replaced with a chondrogenic medium, supplemented with chondrocyte growth factors, and maintained for 14 days.

After 14 days in static culture, half of the scaffolds were attached to the culture chambers of the bioreactor (Electroforce BioDynamic 5210, Bose/TA Instruments) with 180 mL of DMEM medium, supplemented with 10% FBS and antibiotics at 37 °C in a 5% CO_2_ atmosphere. The medium in the bioreactor was pumped with a continuous dynamic flow rate of 0.4 mL min-1.^20^ The bioreactor chambers were configured in a dynamic flow mode, in which the culture medium is actively pumped through the scaffolds via two aligned columns. Due to the tight positioning of the scaffolds between these columns, the system enables direct perfusion of the medium through the scaffold structure. The other half was maintained in static culture for the control group. After 21 days, the cells in the scaffolds, both from static culture and those maintained in the bioreactor, were fixed with 4% paraformaldehyde (PFA) (Sigma-Aldrich, #8.18715), and the cell nuclei were stained with Fluoroshield with DAPI (Sigma-Aldrich, #F6057). Indirect immunostaining was also performed on the samples to analyze the expression of aggrecan, collagen II, and SOX9. The antibodies used in this assay were #ab3778, #ab185430, #ab150115 and #ab185966 (Abcam, Cambridge, MA, USA). The results were analyzed using laser scanning confocal microscopy with a Zeiss LSM 710 system (Carl Zeiss Microscopy GmbH, Jena, Germany). After image acquisition, the aggrecan, collagen II, and SOX9 expressions were quantified.

### 2.8 Fluorescence Profile Analysis

Line profile fluorescence data were analyzed in OriginPro (OriginLab). For each marker (Type II collagen, aggrecan and SOX9), the area under the curve (AUC), obtained by numerical integration of the fluorescence intensity profile, peak intensity (y_0_), and full width at half-maximum (fwhm) were extracted. Because these parameters exhibit different numerical scales, values were normalized to a 0–1 range using min–max scaling across all groups prior to generating the radar plot. Normalization was applied exclusively for graphical visualization.

### 2.9 Statistical Analysis

Statistical analysis was conducted to assess significant differences, whereby *p* < 0.001 was regarded as highly significant and *p* < 0.05, *p* < 0.01 as significant. One-way ANOVA with Tukey’s post hoc test was employed for multiple group comparisons.

## 3 Results and Discussion

### 3.1 Scaffolds Printing

The PLDLA-TMC scaffolds in the 60/40 ratio obtained from printing at a temperature of 120 °C are shown in Figure 1.

**Figure 1:**
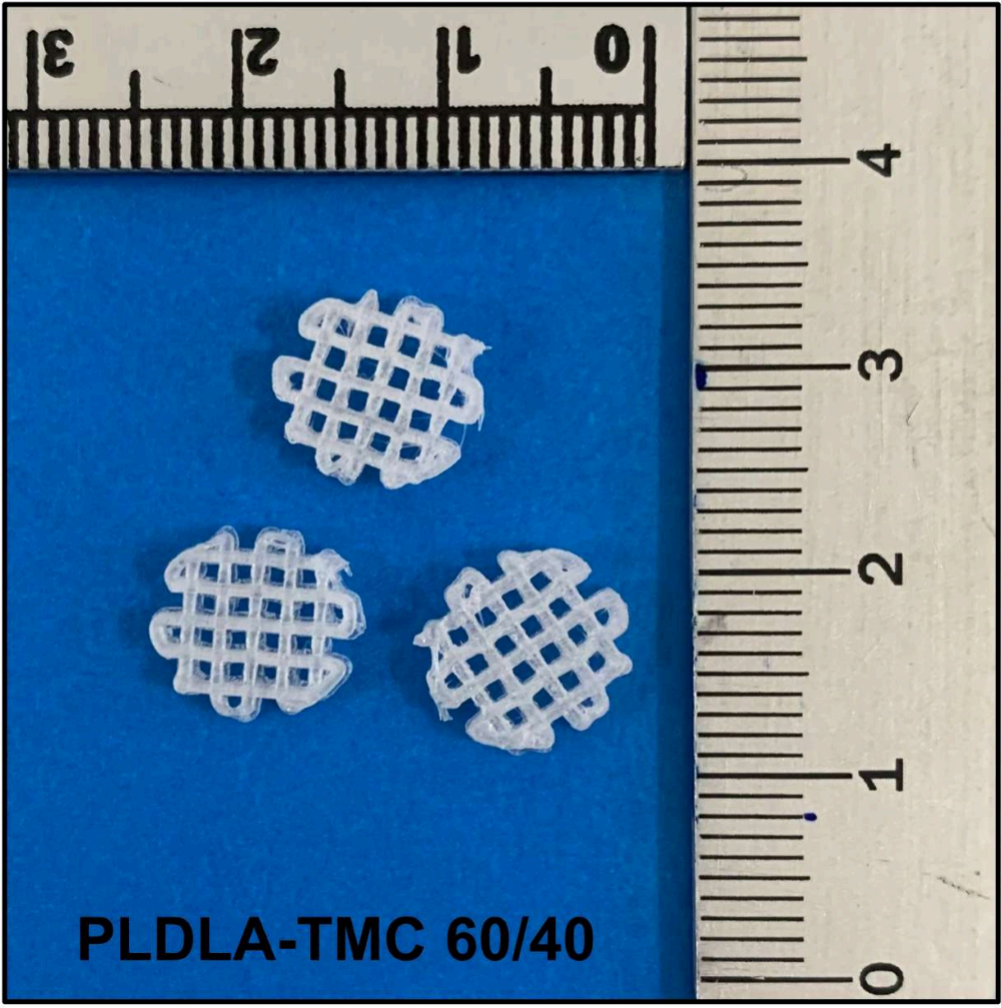
3D-printed scaffolds of PLDLA-TMC 60/40.

### 3.2 Gel Permeation Chromatography (GPC)

The variations in weight-average molecular weight (*M*_w_), number-average molecular weight (*M*_n_), and polydispersity index (PI) of the PLDLA-TMC 60/40 scaffolds as a function of printing temperature are summarized in Table 1.

**Table 1:** GPC Analysis^a^

Printing temperature (°C)	*M*_n_ (g mol^–1^) (10^3^)	*M*_w_ (g mol^–1^) (10^3^)	PI
120	91	194	2.12
130	74	143	1.91
140	72	143	1.98
150	68	118	1.72
160	63	116	1.84
170	57	108	1.89
180	53	104	1.94
190	48	94	1.92

a *M*_n_, *M*_w_, and PI for the PLDLA-TMC 60/40 scaffolds printed at different temperatures.

The molecular weight analysis of the PLDLA-TMC 60/40 scaffolds printed at different temperatures revealed a direct correlation between printing temperature and material degradation. A progressive increase in printing temperature resulted in a reduction in both *M*_n_ and *M*_w_ values. Specifically, printing the scaffolds at 120 °C led to an average decrease of 35% in *M*_n_ and *M*_w_ compared to the preprinting values (*M*_n_: 163,411 g mol^–1^; *M*_w_: 257,919 g mol^–1^). At higher temperatures (180 and 190 °C), degradation became more pronounced, resulting in an approximate 70% reduction in *M*_n_ and *M*_w_.

This effect is attributed to thermomechanical degradation, in which elevated temperatures intensify the degradation induced by shear stresses during the printing process and is consistent with the behavior of aliphatic polyesters subjected to thermomechanical stresses during extrusion-based processes.^21,22^ Shear stress promotes chain scission, while increased thermal energy accelerates the cleavage of covalent bonds along the polymer backbone. As a result, a significant reduction in molecular weight is observed.^23,24^ In addition to changes in *M*_n_ and *M*_w_, variations in the polydispersity index (PI) suggest a nonuniform degradation process, which can further compromise the scaffold’s mechanical properties.^25,26^ The selection of the printing temperature must therefore consider the implications of molecular weight reduction, as this parameter directly influences not only the mechanical strength but also the dimensional stability and structural integrity of the scaffold during cell culture or implantation, since excessive degradation can limit the duration of mechanical support required for effective tissue regeneration.^27,28^

These results underscore the need to define an optimal processing window, which must balance sufficient thermal softening for printability with the preservation of molecular weight to maintain the desired mechanical and biological performance. Temperatures beyond this range may undermine the material’s structural fidelity and functional applicability in tissue engineering applications.^29,30^

### 3.3 Carbonyl Index

The polymer chains breaking during the thermomechanical degradation of poly­(lactic acid)-based (PLA) polymers is associated with the formation of carbonyl groups (C=O), resulting in variations in the intensity of the peak at 1750 cm^–1^ in the FT-IR spectrum.^31,32^ In order to assess this effect, the carbonyl index of PLDLA-TMC 60/40 scaffolds printed at different temperatures was calculated. The index was determined by the ratio between the maximum intensity of the C=O peak at 1750 cm^–1^ and the C–H peak at 1453 cm^–1^, as shown in Figure 2.

**Figure 2:**
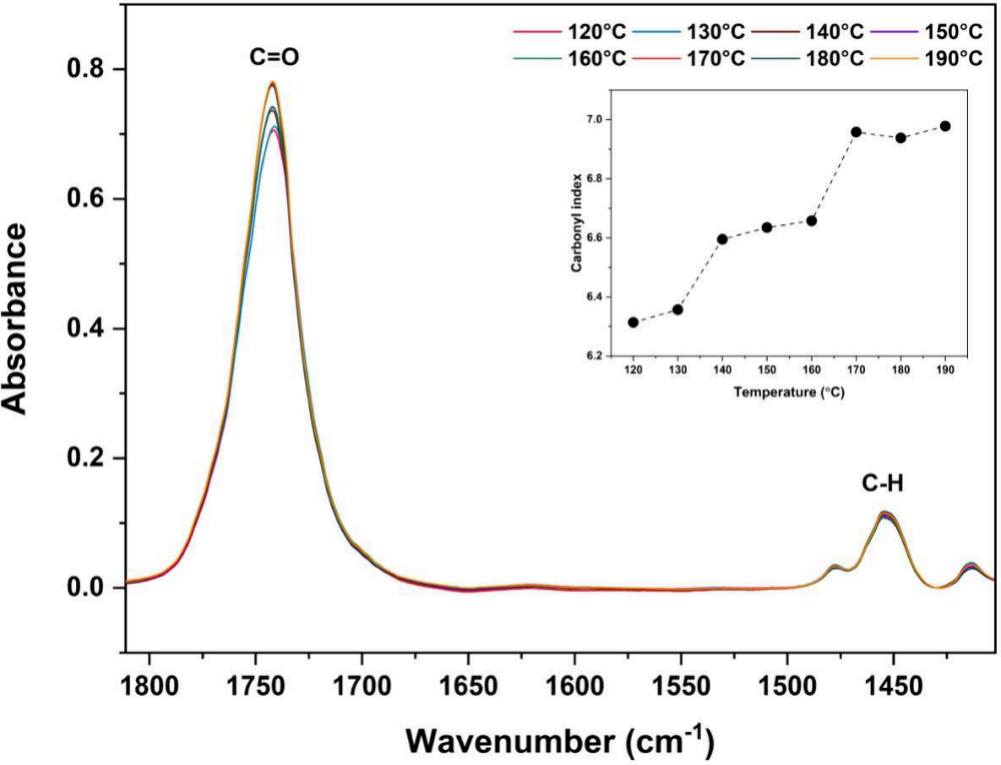
Carbonyl index of PLDLA-TMC 60/40 by FT-IR spectra. The graph presents spectra of the C=O and C–H bands and the corresponding curve of the carbonyl index variation as a function of the printing temperature for the PLDLA-TMC 60/40 scaffolds.

The analysis of the carbonyl index of PLDLA-TMC 60/40 scaffolds printed at different temperatures revealed a direct correlation between increasing carbonyl formation and rising printing temperatures. This behavior reflects the progressive thermomechanical degradation of the polymer, as higher temperatures facilitate chain scission and the consequent generation of carbonyl-containing byproducts.^33^ The comparison between the boundary temperatures (120 and 190 °C) showed an increase in the carbonyl index from 6.3 to 6.9, reinforcing the degradation pattern previously observed in the molecular weight analysis. Therefore, printing at 120 °C emerges as a more suitable condition for maintaining the structural stability of PLDLA-TMC, reducing both molecular degradation and the formation of degradation-related functional groups. Moreover, the presence of carbonyl groups may impact not only the structural stability but also the surface properties of the scaffold, potentially influencing cell-material interactions and accelerating hydrolytic degradation. This acceleration is attributed to the increased polarity of the polymer chains induced by carbonyl groups, which enhances water absorption and facilitates hydrolytic cleavage of ester bonds.^34^

#### 3.4 Differential Scanning Calorimetry (DSC)

The DSC curves corresponding to the second heating ramp of the PLDLA-TMC 60/40 scaffolds printed at different temperatures are shown in Figure 3.

**Figure 3:**
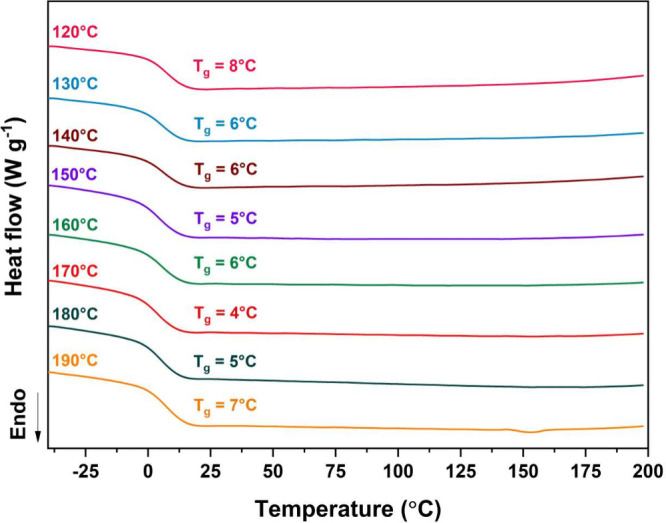
DSC curves. The second heating ramp of PLDLA-TMC 60/40 scaffolds printed at different temperatures.

The thermal analyses identified only the glass transition temperature (*T*_g_), confirming the amorphous nature of the scaffolds, even after the printing process. As reported by Pedrini et al. (2024),^9^ the PLDLA-TMC terpolymer at a 60/40 ratio exhibits a *T*_g_ around 5 °C prior to printing. Consistently, the scaffolds printed at different temperatures did not show a significant shift in *T*_g_ values (Figure 3). These findings suggest that the printing process did not compromise the amorphous structure of the material, preserving its thermal behavior within the expected range for this terpolymer composition. This observation is in agreement with previous studies.^18,35^ In addition, it is worth noting that the absence of *T*_g_ variation, even in the presence of molecular weight reduction observed in the GPC analysis, is consistent with the behavior described for other amorphous polyesters subjected to thermomechanical processing during 3D printing. As reported in the literature, chain scission and transesterification reactions induced by thermal and shear stresses primarily affect the molecular weight distribution and polydispersity, rather than altering the polymer’s amorphous nature or its thermal transition temperatures.^36,37^

### 3.5 Thermogravimetric Analysis (TGA)

The thermal stability of PLDLA-TMC 60/40 scaffolds printed at different temperatures was evaluated by thermogravimetric analysis (TGA), as shown in Figure 4. The TG derivate (DTG) curves of the PLDLA-TMC 60/40 scaffolds revealed two main mass loss events for all the printing temperatures evaluated. The first, less pronounced event, corresponding to an average mass loss of approximately 12% between 100 and 225 °C, is likely associated with the release of absorbed moisture and low-molecular-weight byproducts formed during thermomechanical degradation in the printing process. As detailed in Table 2, the second and more significant mass loss, around 85%, was recorded between 240 and 300 °C, with two consecutive maximum decomposition stages (*T*_max_) observed for all samples. Notably, increasing the printing temperature led to a reduction in both the onset temperature of thermal decomposition (T_onset_) and the *T*_max_. This behavior is indicative of the structural degradation of the polymer, corroborating the molecular weight reduction identified by GPC and the higher carbonyl content revealed by FT-IR.^9^

**Figure 4:**
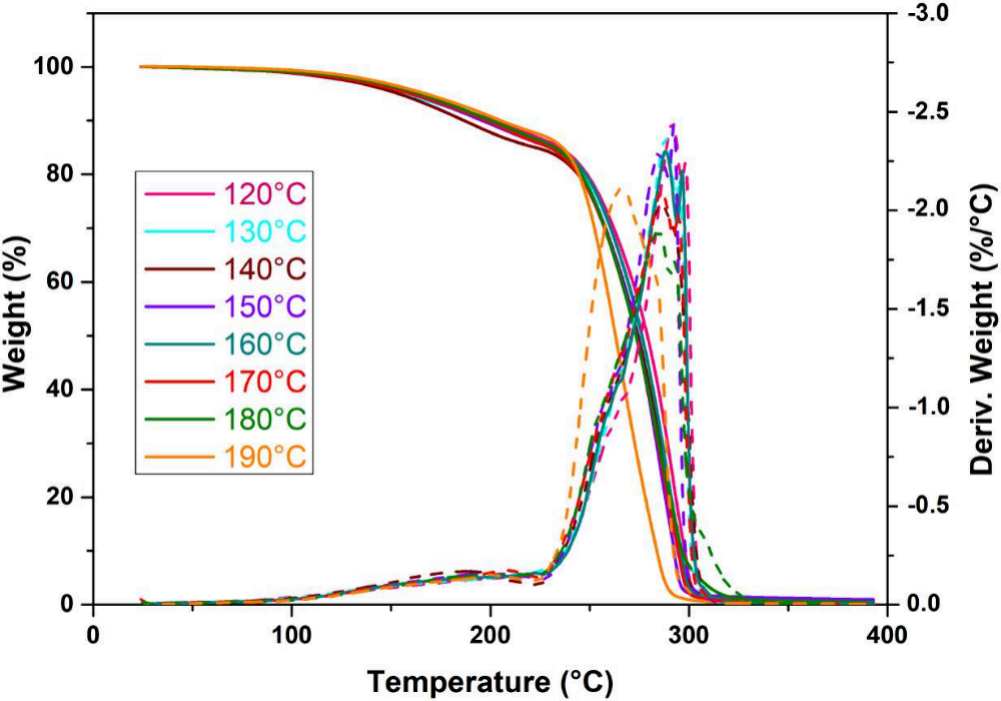
Thermogravimetric analysis. TG and DTG curves of PLDLA-TMC 60/40 scaffolds printed at different temperatures.

**Table 2:** Thermal Properties^a^

Printing temperature (°C)	*T*_onset_ (°C)	*T*_max_ (°C)	Weight loss (%)
120	250	291/297	86
130	252	288/295	85
140	248	285/291	85
150	248	284/291	85
160	249	287/296	86
170	246	285/294	86
180	245	284/293	86
190	243	265/282	88

a The second decomposition event of PLDLA-TMC scaffolds printed at different temperatures.

The observed changes in thermal behavior can be attributed to the scission of covalent bonds during thermomechanical degradation, which generates free radicals capable of initiating further degradation cascades, consequently lowering both T_onset_ and *T*_max_. Thermomechanical degradation during printing generates compounds with reduced thermal stability, which are eliminated at different stages of thermal analysis. Additionally, the presence of oxidation products such as esters and carbonyl groups contributes to the accelerated thermal instability typically observed in poly­(lactic acid)-based polymers during the heating process.^31,38–40^

### 3.6 Hydrolytic Degradation

Having established the influence of the printing temperature on the structural, chemical, and thermal stability of PLDLA-TMC 60/40 scaffolds, the next step was to evaluate the material’s behavior under hydrolytic degradation conditions, which are critical for predicting its long-term performance in biological environments.

Temperature was identified as the main factor influencing the material properties throughout the previous analyses. Therefore, the selection of printing conditions must balance the preservation of the material’s structural and chemical characteristics with the feasibility of the printing process. Among the evaluated conditions, the printing temperature of 120 °C was defined as optimal, as it resulted in the least impact on the material’s chemical integrity and thermal stability. Based on this condition, scaffolds printed at 120 °C were subjected to hydrolytic degradation testing. The evaluation periods were defined as 2 and 4 weeks, with the 4-week point aligned with the subsequent dynamic culture assay.

After 2 and 4 weeks of hydrolytic degradation, the samples were weighed to quantify time-dependent mass loss. As shown in Figure 5, the scaffolds exhibited an average reduction of approximately 2% after 2 weeks, which further increased to about 8% after 4 weeks. One-way ANOVA revealed a significant effect of degradation time on mass loss. Tukey’s post hoc test indicated that no significant difference was observed between the initial condition (0 weeks) and 2 weeks (*p* > 0.05), whereas the 4-week group showed a highly significant increase in mass loss compared to both 0 weeks (*p* < 0.001) and 2 weeks (*p* < 0.01). The results suggest a progressive degradation profile, which is in agreement with the expected hydrolysis behavior of PLDLA-TMC-based scaffolds under physiological-like conditions, where hydrolytic cleavage of ester bonds leads to a gradual loss of mass over time.^41^

**Figure 5:**
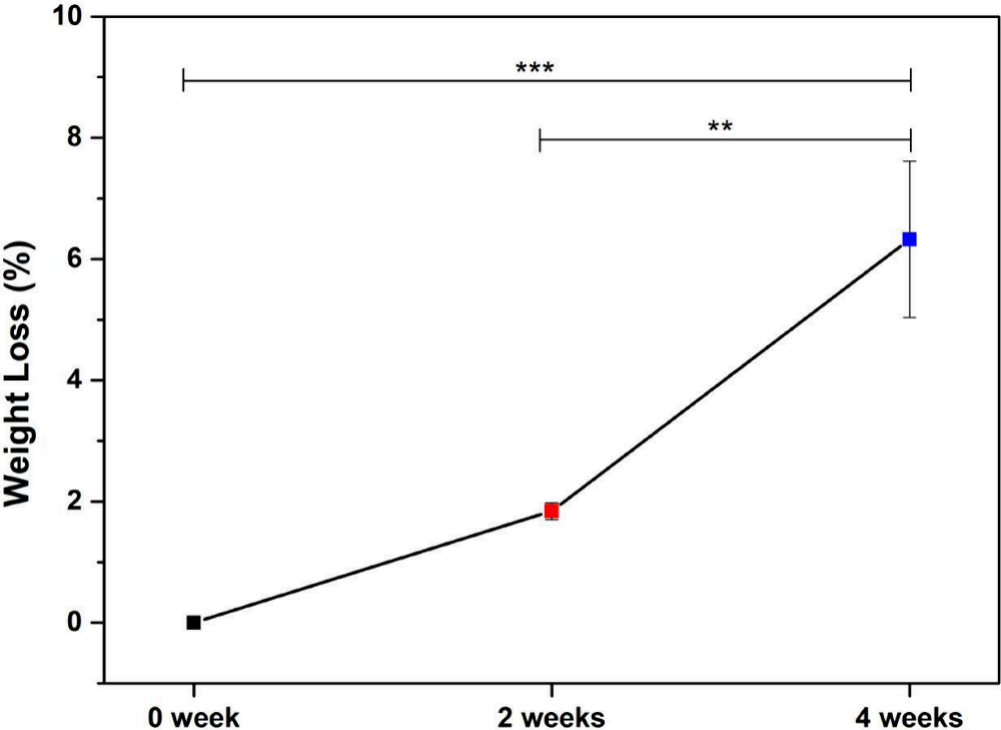
Degradation hydrolytic assay. Weight loss of PLDLA-TMC 60/40 scaffolds printed at 120 °C after being subjected to hydrolytic degradation for 0, 2, and 4 weeks. (Data are presented as mean ± SD with ***p < 0.001; **p < 0.01.)

Additionally, although the observed degradation profile suggests a potentially favorable behavior for biomedical applications, this indication remains preliminary, as the current analysis was limited to mass loss under static *in vitro* conditions. A gradual reduction in mass is generally desirable, as it allows the scaffold to maintain its structural role during the initial stages of cell proliferation while progressively resorbing to enable tissue ingrowth. This balance between stability and degradability is considered essential in tissue engineering strategies.^42^ Nevertheless, it is important to emphasize that the degradation assay performed in this study does not fully replicate the complex dynamics of physiological environments, where enzymatic activity, cellular interactions, and fluid flow can significantly influence the degradation process.

### 3.6.1 Gel Permeation Chromatography (GPC)

After the hydrolytic degradation test at 0, 2, and 4 weeks, the molecular weight of PLDLA-TMC 60/40 scaffolds printed at 120 °C was evaluated by GPC, as shown in Table 3.

**Table 3:** GPC Analysis^a^

Degradation time	M_n_ (g mol^‑1^) (10^3^)	M_w_ (g mol^‑1^) (10^3^)	PI
0 week	258	334	1.29
2 weeks	105	218	2.06
4 weeks	36	87	2.42

a Mean values of *M*_n_, *M*_w_, and PI of PLDLA-TMC 60/40 scaffolds printed at 120 °C after being subjected to hydrolytic degradation for 0, 2, and 4 weeks (*n* = 3).

A marked reduction in molecular weight was observed as degradation progressed. After 2 weeks, both *M*_n_ and *M*_w_ decreased by approximately 50% compared to the nondegraded control (week 0), with statistical analysis confirming significant differences between the groups (*p* < 0.01). This reduction was accompanied by an increase in the PI, indicating a broadening of the molecular weight distribution consistent with a random chain scission degradation mechanism. After 4 weeks, the decrease in molecular weight became even more pronounced, reaching an approximate 80% reduction relative to the initial value. These changes were statistically significant when compared with both 0 weeks (*p* < 0.001) and 2 weeks (*p* < 0.01), further supporting the progressive hydrolytic fragmentation of polymer chains over time (Figure 6).

**Figure 6:**
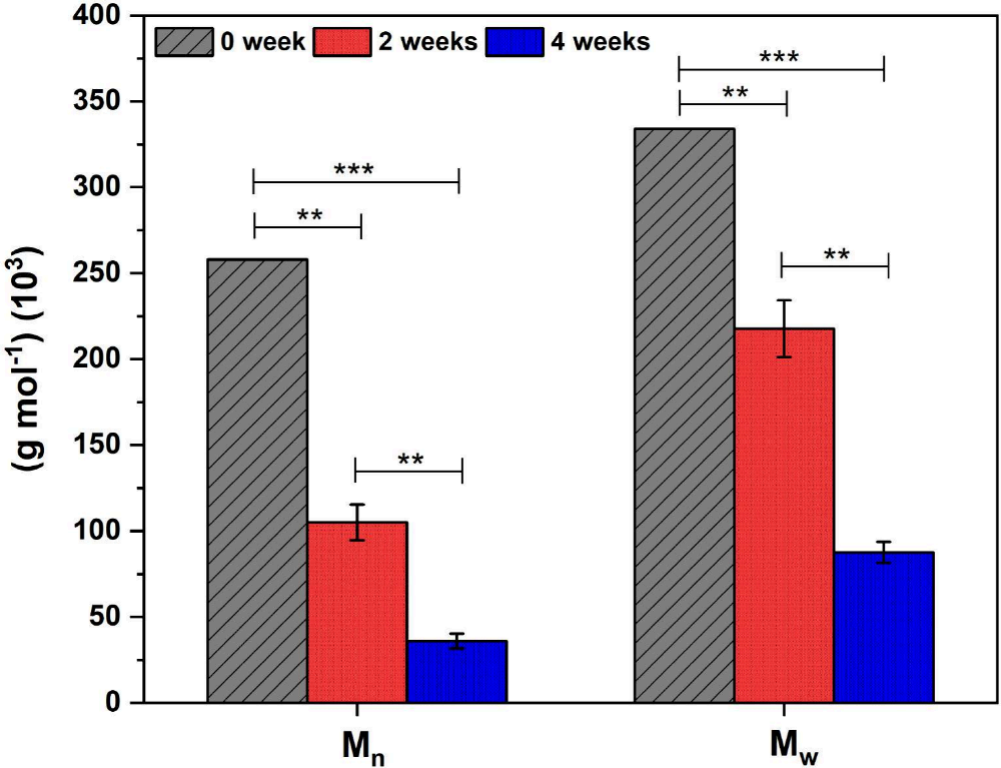
Mean *M*_n_ and *M*_w_ values (±SD) of PLDLA-TMC 60/40 scaffolds after 0, 2, and 4 weeks of hydrolytic degradation. Results are based on triplicate measurements (*n* = 3) with **p < 0.01 and ***p < 0.001.

Despite the significant molecular weight reduction, the total mass loss of the scaffolds remained limited during the same period (as shown in Figure 5). This discrepancy is commonly observed in hydrolytically degradable polyesters, as the cleavage of polymer chains initially leads to soluble oligomers and monomers, such as lactic acid, which gradually diffuse out of the polymer matrix without an immediate impact on the bulk mass of the sample.^43^

### 3.6.2 Carbonyl Index

The carbonyl index, used to assess the impact of hydrolytic degradation on PLDLA-TMC 60/40 scaffolds printed at 120 °C after 0, 2, and 4 weeks is presented in Figure 7.

**Figure 7:**
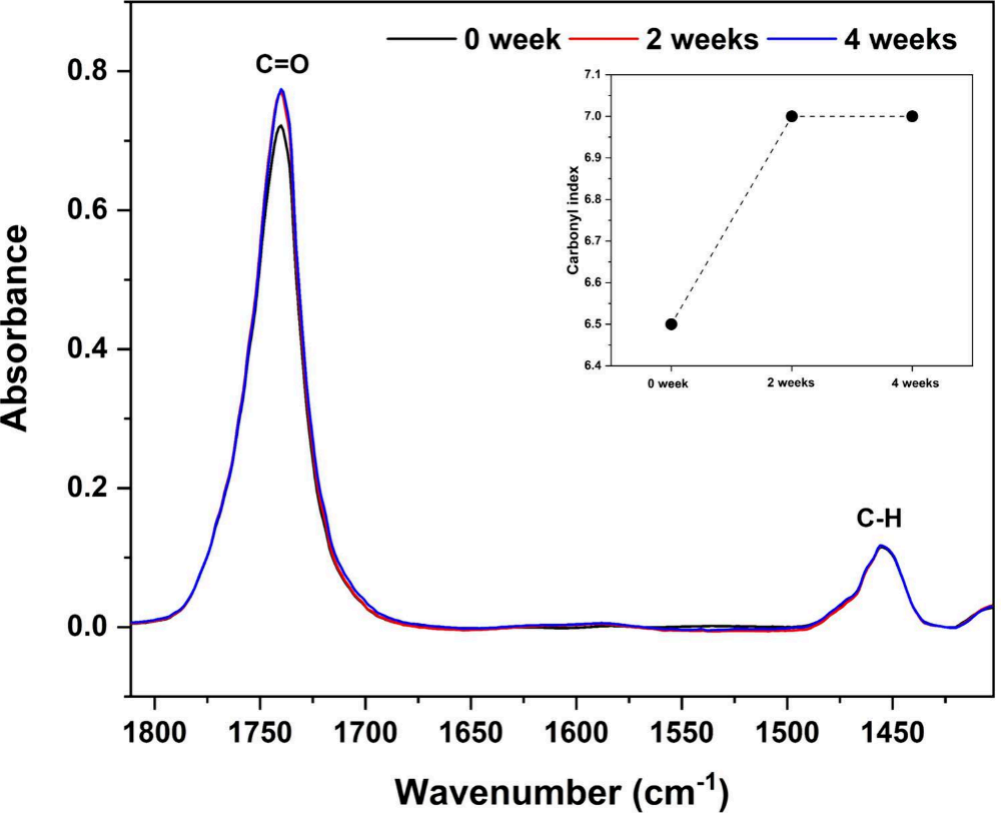
Carbonyl index by FT-IR spectra. The graph shows spectra of the C=O and C–H bands, and the corresponding curve of the carbonyl index variation for the PLDLA-TMC 60/40 scaffolds printed at 120 °C and subjected to hydrolytic degradation for 0, 2, and 4 weeks.

As observed, the carbonyl index increased progressively after 2 and 4 weeks of exposure to the hydrolytic medium when compared to the nondegraded samples (week 0). This increase, detected through FT-IR analysis, indicates the formation of carbonyl groups, which are characteristic of the hydrolytic cleavage of ester bonds, suggesting the progression of the degradation process.^33^ Interestingly, the carbonyl index remained practically unchanged between the 2- and 4-week time points. According to Rowe et al. (2016),^44^ the hydrolytic degradation of polyesters can differ between the surface and the bulk of the material, as the diffusion of degradation products is strongly influenced by the dimensions of the specimen. In larger samples, the limited diffusion of acidic degradation products may lead to their accumulation within the inner regions of the scaffold, accelerating autocatalytic hydrolysis in the bulk, while surface degradation tends to stabilize. Considering that the FT- IR technique analyzes only the superficial layer of the material (typically between 1 and 2 μm in depth), it is likely that, after 2 weeks, the surface degradation had already plateaued, while further degradation progressed predominantly in the inner structure of the scaffold, which may explain the lack of significant variation in the carbonyl index at 4 weeks.

### 3.6.3 Thermal Analysis

PLDLA-TMC 60/40 scaffolds printed at 120 °C and subjected to hydrolytic degradation for 0, 2, and 4 weeks were thermally evaluated by DSC (Figure 8A) and TGA (Figure 8B).

**Figure 8:**
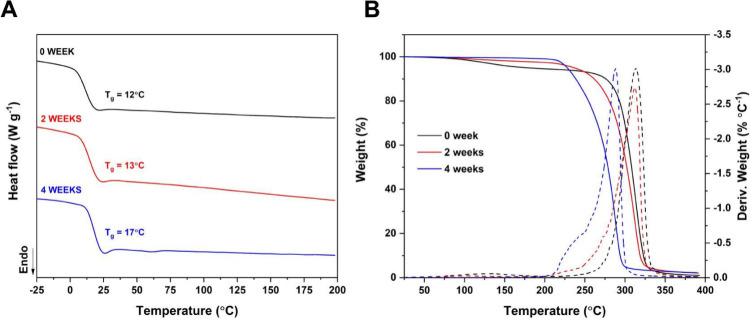
Thermal analysis. (A) DSC and (B) TGA of PLDLA-TMC 60/40 scaffolds printed at 120 °C subjected to hydrolytic degradation for 0, 2, and 4 weeks.

The second DSC heating ramp (Figure 8A) revealed that all samples exhibited only the *T*_g_, confirming their amorphous nature. No significant changes were observed in the *T*_g_ of the samples at 0 weeks (12 °C) and 2 weeks (13 °C). However, after 4 weeks of degradation, a slight increase in *T*_g_ to 17 °C was noted, indicating that the degradation time (4 weeks) affected the polymer structure. Despite this shift, the observed *T*_g_ values remained below body temperature, ensuring that the scaffold will not undergo stiffening during use, preserving its ability to deform, maintain flexibility, and consequently, its potential application as cartilage. Additionally, no events associated with crystallinity formation were observed, which is a crucial characteristic for the intended application. The absence of crystalline phases minimizes the risk of crystalline fragment formation during the degradation process, which could potentially induce an undesired inflammatory response and compromise scaffold effectiveness.^35^

The TGA analysis (Figure 8B) revealed more significant changes in the thermal behavior of PLDLA-TMC 60/40 over the hydrolytic degradation period. The week 0 sample exhibited two distinct mass loss events, as previously described in this study. The first, less prominent, occurred between 100 and 200 °C, while the second, more intense event, marked by a 92% mass loss, had a T_onset_ at 292 °C and a *T*_max_ at 313 °C. However, after 2 and 4 weeks, the samples exhibited a single mass loss event of approximately 96%, accompanied by a shift of these events to lower temperatures. The 2-week sample showed a T_onset_ at 282 °C and a *T*_max_ at 312 °C, whereas the 4-week sample showed more pronounced changes, with a T_onset_ at 242 °C and a *T*_max_ at 290 °C.

These results demonstrate a progressive reduction in thermal stability throughout the hydrolytic degradation process. This behavior aligns with the findings of Komatsu et al. (2024),^35^ who reported similar results during the *in vitro* degradation of 3D-printed PLDLA-TMC scaffolds at a 70/30 ratio. The loss of lower molecular weight fractions in the initial degradation weeks, as evidenced by GPC analysis, likely explains the transition to a single thermal degradation event in the samples exposed to longer degradation periods. Furthermore, the shift of degradation temperatures to lower values suggests a decrease in the material’s thermal resistance, potentially due to polymer chain scission and the formation of structures with lower thermal stability.

### 3.6.4 Scanning Electron Microscopy (SEM)

The SEM images of the PLDLA-TMC 60/40 scaffolds surface printed at 120 °C and subjected to hydrolytic degradation for 0, 2, and 4 weeks are shown in Figure 9.

**Figure 9:**
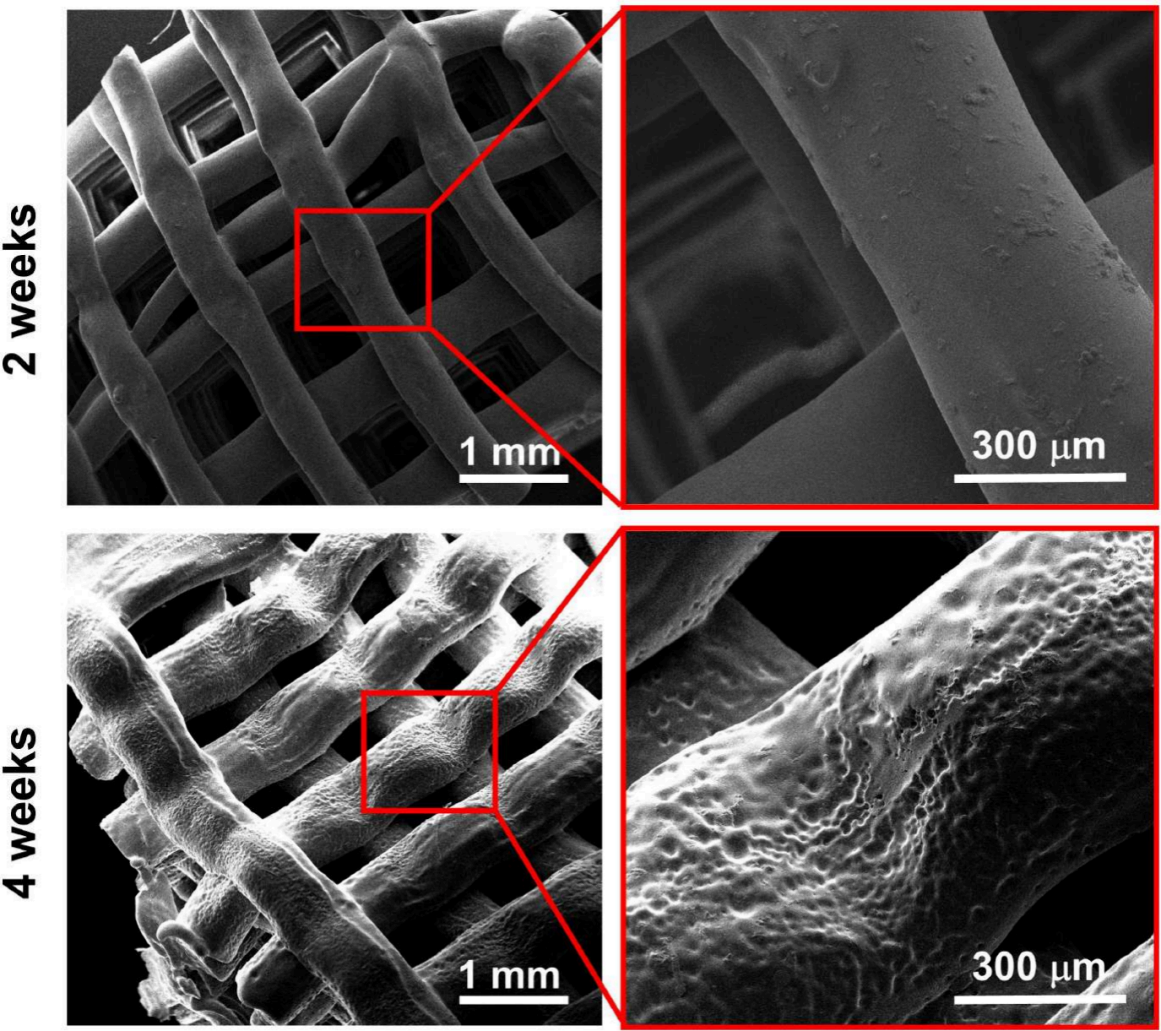
SEM micrographs. Images of PLDLA-TMC 60/40 scaffolds printed at 120 °C subjected to hydrolytic degradation for 2 and 4 weeks at different magnifications.

These images provided valuable insights into the surface morphology of the scaffolds throughout the degradation process. After 2 weeks of hydrolytic exposure, the scaffolds retained a smooth, interconnected filamentous structure with a relatively uniform pore distribution. This suggests that, during the initial stages of degradation, the scaffold maintains its structural integrity, supporting the idea that PLDLA-TMC scaffolds may provide a favorable environment for cell proliferation and tissue ingrowth, which is crucial for tissue engineering applications.^45^

However, after 4 weeks of degradation, significant morphological alterations were evident. The filaments displayed erosion across the scaffold’s surface, accompanied by the formation of surface grooves. These grooves are indicative of polymer chain fragment dissolution, a direct consequence of the hydrolysis process. This phenomenon is in line with the observations from the GPC analysis, which showed a reduction in the molecular weight of the PLDLA-TMC scaffolds over time.^44^ The erosion of the filaments could also be associated with the hydrolytic medium’s absorption, leading to fiber swelling, which was observed in the SEM images. This swelling, coupled with the erosion, suggests that the scaffold is undergoing significant degradation, a trend confirmed by the decrease in thermal stability observed in the TGA and DSC analyses.

The observed changes in surface morphology can have important implications for the scaffold’s performance. For instance, the erosion and groove formation could increase the scaffold’s surface area, which may enhance cellular attachment and tissue infiltration over time. This is particularly important in the context of cartilage tissue engineering, where scaffold degradation must occur at a rate that allows for proper tissue regeneration without compromising mechanical support during the early stages of healing.^35^ This balance between structural degradation and tissue regeneration must be carefully managed for optimal performance in biomedical applications, as previously highlighted by Sabir et al. (2009).^45^

A previous study conducted a comprehensive physicochemical and biological characterization of PLDLA-TMC 60/40 prior to 3D printing.^9^ The results demonstrated that this terpolymer composition, along with the selected printing parameters, decisively influences the mechanical, thermal, and degradation properties of PLDLA-TMC scaffolds. Therefore, the present study builds upon those established findings and does not include an unprinted control or a PLDLA-only reference material, as these controls were extensively characterized in that earlier work.

In conclusion, the SEM analysis, in conjunction with other characterization techniques, offers a comprehensive understanding of how PLDLA-TMC scaffolds degrade over time under hydrolytic conditions. The progressive erosion and surface modification observed at the 2 and 4-week time points suggest that while the material undergoes degradation, it retains enough structural integrity to facilitate tissue ingrowth during the early stages of scaffold use. These outcomes provide a solid foundation for conducting dynamic culture assays in bioreactors, which will allow the evaluation of cell-scaffold interactions in a more physiological environment with flow conditions, essential for more accurately simulating the material’s behavior in biological systems.

### 3.7 Bioreactor Dynamic Culture

The 3D-printed PLDLA-TMC 60/40 scaffolds were initially maintained under static culture conditions for 14 days to allow MSC differentiation. Subsequently, the scaffolds were transferred to a bioreactor system and cultured under dynamic perfusion conditions for an additional 21 days before fixation and analysis.

Laser scanning confocal microscopy was employed to assess the expression of aggrecan, type II collagen, and SOX9, key markers involved in cartilage extracellular matrix composition and chondrogenic differentiation. The scaffolds cultured under dynamic conditions were compared to those maintained under static conditions for the same total period (Figures 10A and 10B).

**Figure 10:**
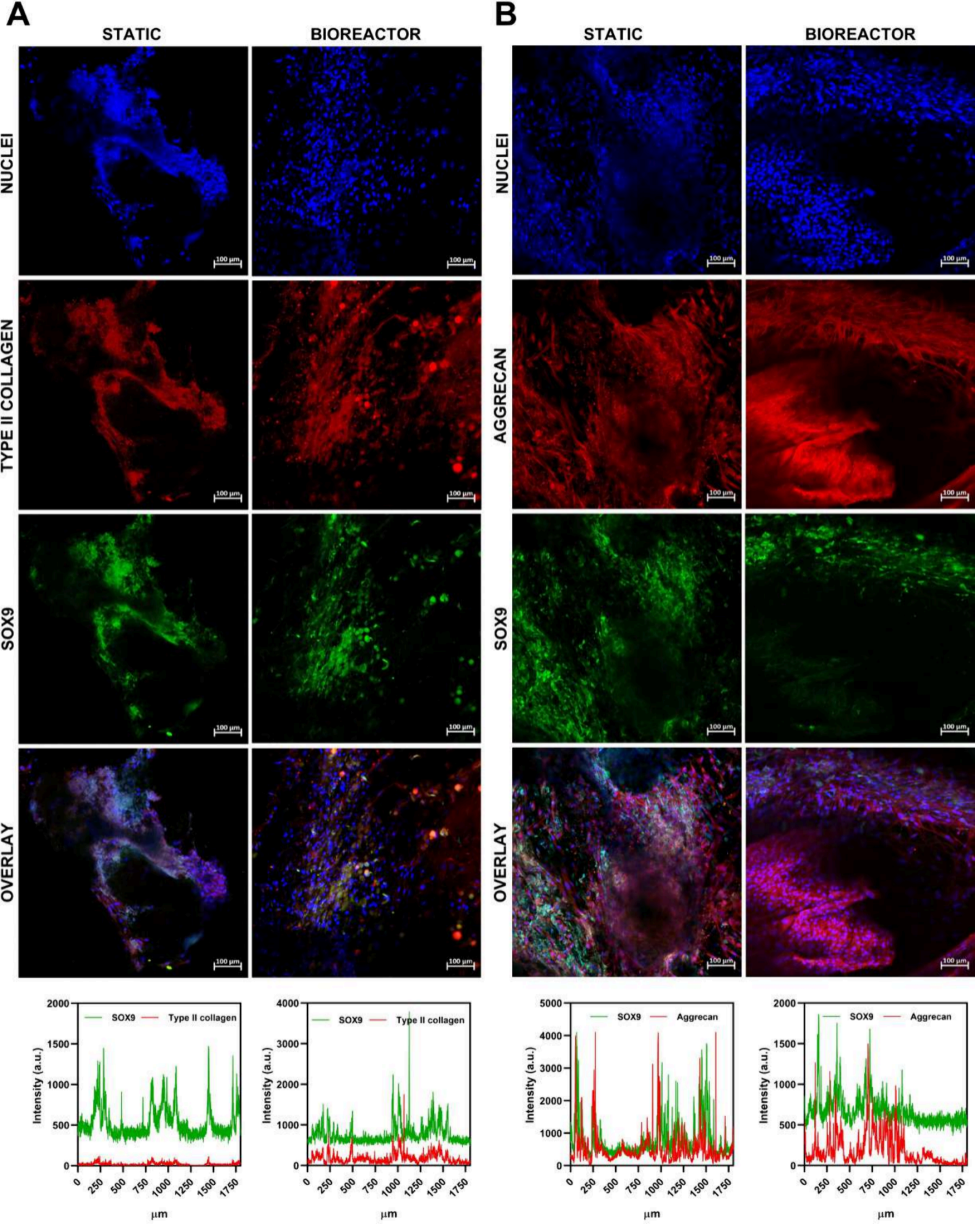
Confocal laser scanning micrographs of MSCs differentiated into chondrocytes after 21 days of culture under static or dynamic conditions. Both panels correspond to the same culture period and culture conditions, differing only in the chondrogenic marker analyzed, both labeled with the same secondary antibody conjugated red fluorophore. (A) DAPI, type II collagen, SOX9, merged images, and fluorescence intensity profiles. (B) DAPI, aggrecan, SOX9, merged images, and fluorescence intensity profiles.

The micrographs demonstrated successful MSC differentiation into chondrocytes within the PLDLA-TMC 60/40 scaffolds, as evidenced by the expression of all three markers.^46,47^ Notably, Figure 10A illustrates an increased fluorescence intensity for collagen type II and SOX9 in the dynamically cultured scaffolds compared to the static ones. This observation reflects the higher cell density and more homogeneous cell distribution promoted by perfusion in the bioreactor. Visual inspection of the confocal images suggests that, under static conditions, cells tended to remain localized at the peripheral regions of the scaffold, whereas under dynamic culture, cellular nuclei were observed at greater depths, reaching up to approximately 350 μm. In addition to improved cellular penetration, the bioreactor culture condition significantly enhanced the expression pattern of aggrecan, as shown in Figure 10B. The dynamic culture induced a stratified organization of the newly formed extracellular matrix, resembling the zonal arrangement typical of native cartilaginous tissue. In contrast, the static culture exhibited a more diffuse expression of ECM components. This difference can be attributed to the improved nutrient and oxygen diffusion promoted by perfusion flow, which created a more favorable environment for cell proliferation and differentiation.^48^ It is important to emphasize that, during the dynamic culture period, the cells were maintained in a differentiation medium devoid of external chondrogenic supplements. The mechanical stimuli generated by the flow, combined with paracrine signaling from already differentiated cells, proved sufficient to sustain chondrogenesis, confirming the pivotal role of mechanical and microenvironmental cues in the regulation of stem cell fate.^49^

Moreover, the quantification of SOX9 expression corroborated the differentiation process in both conditions. As a master transcription factor in chondrogenesis, SOX9 plays a dual role, initially maintaining MSC proliferation while simultaneously promoting the expression of cartilage-specific genes. Its persistent expression throughout the culture period supports the successful transition from undifferentiated MSCs to mature chondrocytes.^50^ The data emphasize how scaffold properties, the mechanical environment, and cellular behavior are tightly interconnected. The previously observed degradation-induced morphological modifications, such as increased surface roughness and the formation of grooves during hydrolytic exposure, may have contributed to enhancing cell attachment and migration within the scaffold. This synergy between scaffold degradation, architecture, and mechanical stimulation demonstrates the material’s potential for cartilage tissue engineering applications, where the balance between scaffold resorption, matrix deposition, and cell colonization is essential for successful tissue regeneration.^51^

Fluorescence signals were quantitatively evaluated through profile analysis of confocal images. The area under the curve (AUC), peak intensity (y_0_), and full width at half-maximum (fwhm) were extracted to assess protein abundance and spatial distribution, respectively. As shown in Figure 11, dynamic perfusion markedly enhanced chondrogenesis: type II collagen and SOX9 exhibited approximately 2-fold higher AUC and y_0_ values compared to static culture, while their fwhm decreased by ∼ 40–50%, indicating a more localized and organized ECM.^52^ Although aggrecan showed a slightly higher AUC under static conditions (∼10–20% difference), the bioreactor culture reduced its fwhm by ∼ 40%, suggesting superior structural organization consistent with hyaline cartilage formation.^53^ These findings reinforce the enhanced matrix stratification observed in Figure 10 and confirm the beneficial role of dynamic mechanical stimulation in promoting functional ECM deposition.

**Figure 11:**
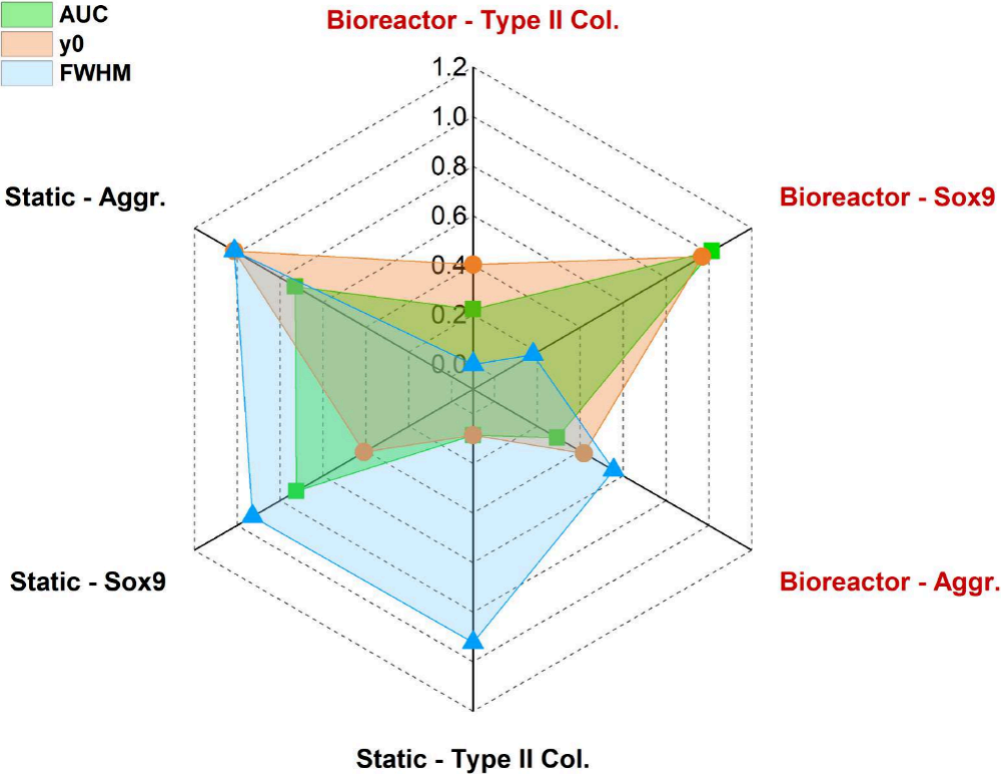
Normalized fluorescence parameters (AUC, y_0_ and fwhm) of chondrogenic markers in static and bioreactor cultures.

In addition, total cell number was estimated based on nuclear fluorescence quantification (Figure 12). The dynamically cultured scaffolds displayed a significantly higher number of nuclei (*p* < 0.05), confirming superior cell viability and infiltration promoted by perfusion. This quantitative evidence supports the hypothesis that nutrient/oxygen transport and mechanical cues generated by flow sustain, cell viability, chondrogenesis, and ECM remodeling.^54–57^

**Figure 12:**
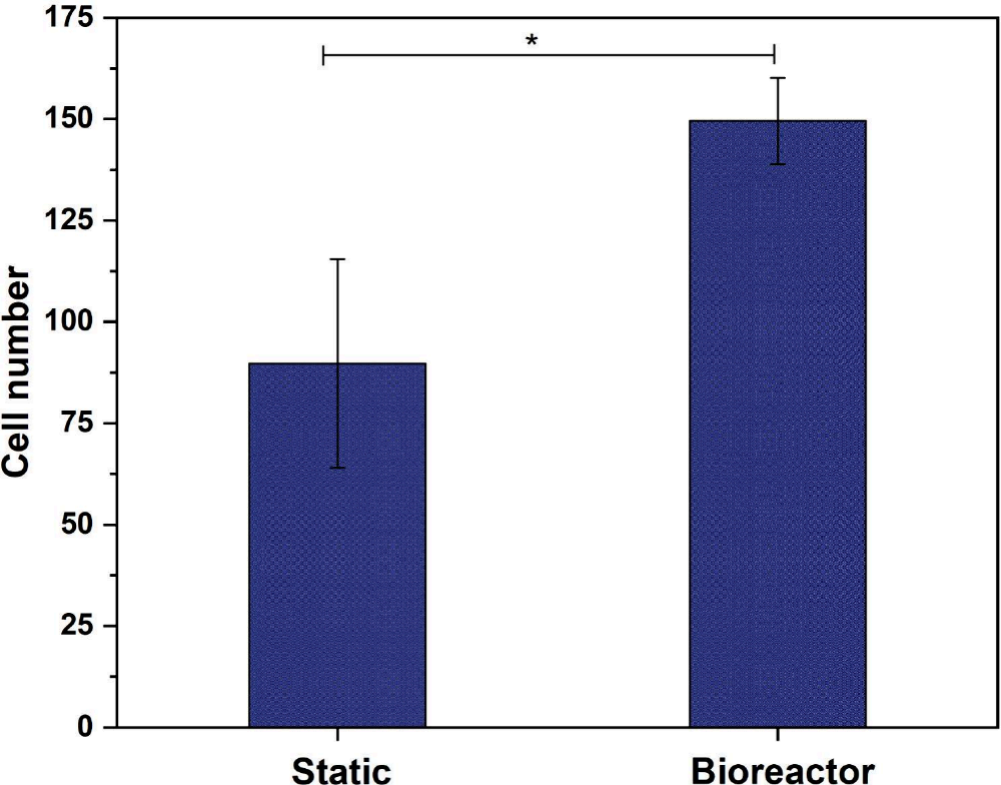
Quantification of cell number in PLDLA-TMC 60/40 scaffolds under static or bioreactor culture. Dynamic perfusion significantly increased cell density. Data are presented as mean ± SD (*n* = 4). Statistical analysis was performed using one-way ANOVA followed by Tukey’s post hoc test (*p < 0.05).

Taken together, the results provide a comprehensive understanding of the structural, physicochemical, and biological behavior of 3D-printed PLDLA-TMC 60/40 scaffolds. The degradation assays demonstrated a progressive, yet controlled, morphological alteration of the scaffold surface over time, marked by filament erosion and the formation of grooves, which not only confirmed the material’s biodegradability but also indicated potential benefits for cellular attachment and migration.

Similar degradation patterns were reported for 3D-printed PLDLA-TMC scaffolds used in meniscal repair, where loss of molecular weight and microstructural alterations were associated with increased cellular adhesion and tissue formation.^35^ In that study, PLDLA-TMC scaffolds seeded with mesenchymal stem cells successfully regenerated meniscal fibrocartilage *in vivo* model after implantation in rabbits, without the need for exogenous growth factors, highlighting how scaffold architecture and biodegradation synergistically support tissue regeneration, as observed in our study. In agreement with these findings, our results show that controlled degradation, together with a porous microarchitecture and biomechanical stimulation, favored cell infiltration, ECM deposition, and zonal organization resembling native cartilage. Therefore, the ability of PLDLA-TMC scaffolds to induce chondrogenic differentiation in a dynamic, growth-factor-free environment represents an important translational advantage, reducing costs, simplifying regulatory pathways, and bringing this technology closer to viable *in vivo* applications.

The relevance of architecture and surface morphology for cartilage-related regeneration is further supported by recent work evaluating a PLDLA–TMC/PVA blend for meniscal repair.^58^ In that study, the scaffold exhibited a porous and intrinsically rough surface, with an average pore size of ∼115 μm and high cross-linking density, which significantly increased material stiffness and was proposed to favor cell proliferation and anchorage. Although the polymeric composition differed from the PLDLA-TMC terpolymer examined here, both studies emphasize that local microstructural cues, rather than biochemical supplementation, play a determining role in guiding cell behavior. In agreement with our findings, the authors demonstrated that the scaffold promoted biocompatibility *in vitro*, indicating that roughness and interconnected porosity can support initial adhesion and subsequent ECM deposition, a critical requirement for functional regeneration of fibrocartilaginous tissues such as the meniscus or articular cartilage.

Although mechanical tests were not conducted in this study, it is important to consider that the observed degradation may also influence the scaffold’s mechanical properties, potentially reducing its load-bearing capacity over time. This consideration is particularly relevant in cartilage tissue engineering, where scaffolds are expected to maintain sufficient mechanical integrity throughout the early stages of regeneration. Studies have shown that PLDLA-based scaffolds, despite undergoing gradual hydrolytic degradation, can retain adequate mechanical strength during the initial phases of implantation, aligning with the temporal requirements for tissue support and remodeling.^59,60^ Under similar degradation conditions, Ciambelli et al. (2013)^59^ showed that after 4 weeks of hydrolytic degradation, PLDLA retained approximately 40% of its initial mechanical properties (Young̀s modulus and elongation). Despite this residual capacity, the material became significantly more fragile and structurally unstable. In our study, however, the presence of flow-induced mechanical stimulation promoted extracellular matrix deposition, which may progressively compensate for the scaffold’s mechanical loss, supporting long-term functionality.^49,61,62^

The potential of PLDLA-TMC 60/40 scaffolds as promising candidates for cartilage tissue engineering is reinforced by these data, demonstrating the importance of integrating material design, degradation kinetics, and dynamic culture strategies to achieve functional tissue regeneration.

## 4 Conclusion

This study aimed to characterize 3D-printed PLDLA-TMC 60/40 scaffolds at different temperatures, focusing on their potential application as cartilage substitutes. The scaffolds were evaluated regarding their physicochemical and thermal properties, hydrolytic degradation profile, and biological performance under dynamic perfusion conditions in a bioreactor. GPC analysis revealed that higher printing temperatures resulted in a significant reduction in molecular weight, likely due to thermomechanical degradation induced by shear forces during the printing process, which was further supported by an increase in the carbonyl index. Thermal stability assessments by TGA demonstrated that elevated printing temperatures lowered the T_onset_, affecting the material’s resistance to mass loss. Nevertheless, DSC confirmed that the amorphous nature of the terpolymer was preserved across all printing conditions. Among the tested conditions, printing at 120 °C proved to induce the least impact in the material’s properties, making it more suitable for the intended application. Therefore, scaffolds printed at 120 °C were selected for hydrolytic degradation assays over 0, 2, and 4 weeks. The results revealed mass losses of 2% and 8% at 2 and 4 weeks, respectively, accompanied by a progressive reduction in molecular weight, as evidenced by GPC analysis. The degradation process also led to a marked increase in the carbonyl index, while DSC and TGA analyses indicated substantial alterations in *T*_g_ and thermal stability — factors that are essential for the scaffold’s performance in biomedical contexts. In parallel, SEM images confirmed the presence of surface erosion and the formation of grooves after 4 weeks, supporting the evidence of structural degradation. Finally, the dynamic culture assays demonstrated that cell differentiation is modulated not only by biochemical factors but also by the mechanical and architectural cues provided by the perfusion environment and the scaffold’s morphological and chemical characteristics. The results provide valuable insights into the hydrolytic and thermomechanical stability of 3D-printed PLDLA-TMC 60/40 scaffolds, particularly under conditions that more closely mimic the physiological environment. By integrating extrusion-based 3D printing with dynamic perfusion culture, this study offers a more realistic and comprehensive evaluation of scaffold performance over time. These findings go beyond conventional static degradation assessments by capturing how fabrication-induced degradation and mechanical stimulation affect scaffold behavior *in vitro*. This integrated approach addresses a critical gap in the literature and supports the application of PLDLA-TMC 60/40 in next-generation cartilage tissue engineering strategies, providing a more predictive model of scaffold behavior *in vivo* that requires both structural resilience and biofunctionality under dynamic conditions.
